# In Vitro and In Vivo Studies of Alar-Nasal Cartilage Using Autologous Micro-Grafts: The Use of the Rigenera^®^ Protocol in the Treatment of an Osteochondral Lesion of the Nose

**DOI:** 10.3390/ph10020053

**Published:** 2017-06-13

**Authors:** Gabriele Ceccarelli, Pietro Gentile, Marco Marcarelli, Martina Balli, Flavio Lorenzo Ronzoni, Laura Benedetti, Maria Gabriella Cusella De Angelis

**Affiliations:** 1 Department of Public Health, Experimental Medicine and Forensic, Human Anatomy Unit, University of Pavia, Pavia 27100, Italy; martina.balli01@universitadipavia.it (M.B.); flavio.ronzoni@unipv.it (F.L.R.); laura.benedetti@unipv.it (L.B.); 2 Center for Health Technologies, University of Pavia, Pavia 27100, Italy; 3 Chief of Plastic and Reconstructive Surgery, Catholic University, Tirana 1005, Albania; pietrogentile2004@libero.it; 4 Plastic and Reconstructive Surgery Department, University of Rome “Tor Vergata”, Rome 00173, Italy; 5 Santa Croce Hospital, Unit of Orthopedics and Traumatology of Chieri and Moncalieri, Turin 10024, Italy; dottmarcarelli@gmail.com

**Keywords:** Rigenera^®^ protocol, autologous micro-grafts, chondrocytes, tissue engineering approaches, nasal valve collapse

## Abstract

Cartilage defects represent a serious problem due to the poor regenerative properties of this tissue. Regarding the nose, nasal valve collapse is associated with nasal blockage and persistent airway obstruction associated with a significant drop in the quality of life for patients. In addition to surgical techniques, several cell-based tissue-engineering strategies are studied to improve cartilage support in the nasal wall, that is, to ameliorate wall insufficiency. Nevertheless, there are no congruent data available on the benefit for patients during the follow-up time. In this manuscript, we propose an innovative approach in the treatment of cartilage defects in the nose (nasal valve collapse) based on autologous micro-grafts obtained by mechanical disaggregation of a small portion of cartilage tissue (Rigenera^®^ protocol). In particular, we first analyzed in vitro murine and human cartilage micro-grafts; secondly, we analyzed the clinical results of a patient with pinched nose deformity treated with autologous micro-grafts of chondrocytes obtained by Rigenera^®^ protocol. The use of autologous micro-graft produced promising results in surgery treatment of cartilage injuries and could be safely and easily administrated to patients with cartilage tissue defects.

## 1. Introduction

Osteochondral pathologies may result from trauma or sports injuries or be the consequence of disorders associated with cartilage degeneration, such as chondromalacia and osteoarthritis [1,2]. Irrespective of the etiology of the osteochondral pathology, the loss of cartilage, evidenced in magnetic resonance images (MRI) as a thickening of the cartilage line, causes pain and physical disability [3,4,5]. In addition, regarding nasal cartilage, obstruction at the level of the nasal valve is a common and long-recognized cause of nasal blockage [6,7]. It is estimated that about 60% of nasal obstruction is limited by the lateral nasal wall and internal valve in some way. The surgical treatment of nasal valve collapse has proven to be difficult over the years, either resulting in unfavorable cosmetic results or resulting in persistent airway blockage [8].

In recent years, many efforts have been made in developing complementary strategies aimed at regenerating the damaged cartilage or shortening the natural regeneration process. In addition to standard surgical techniques based on the use of “spreader” grafts to reconstruct the internal nasal valve [9], various approaches have been proposed to stimulate the production of new cartilage. Biostimulation techniques have included physical approaches, such as the use of high-frequency biostimulation during surgery [10] or low-power laser targeting the osteochondral defect [11], and biological approaches, such as autologous chondrocyte implantation [12] and the use of platelet-rich plasma to stimulate the production of new cartilage [13].

Stem cells play a pivotal role in tissue repair and regeneration in response to injury. The finding of stem-like cell populations with multipotent features in various tissues has motivated the use of these cells for regenerative purposes [14,15]. In fact, mesenchymal stem cells are widely studied in tissue engineering therapeutic approaches [16,17,18]. In particular, for the correction of joint defects, even if Mesenchymal stem cells (MSCs) were derived from different mesoderm tissues, the availability of ready-to-use methods for a safe and effective isolation of mesenchymal stem cells during surgery has been a bottleneck for the clinical use of multipotent cells. In addition, the identification of stem-like cells in many other tissues suggests additional uses of autologous micro-grafts preparation and transfer [19]. Tissue engineering approaches aim to develop new strategies in order to improve interfacial interactions between biomaterials and cells, and to design novel surface modifications to control these interactions in diagnostic systems, medical devices, and for tissue-regenerative purposes. In this regard, electrospraying-related technologies, such as electrospinning, have been extensively studied to create scaffolds used in cell proliferation analysis. Several in vitro studies showed that living cells could be directly electrospun successfully on different organic polymers [20,21]. Although these techniques have been used in in vivo animal models, they cannot be still applied to clinical procedures on humans.

The Rigenera^®^ protocol is a novel strategy for tissue mechanical disaggregation, which allows obtaining autologous micro-grafts enriched with stem cells during surgery [22,23]. Compared to enzymatic techniques, mechanical disaggregation has claimed to be faster and safer. So far, the Rigenera^®^ protocol has shown clinical efficacy in the management of complex wounds [24,25] and for the regeneration of the bone in periodontal surgeries [26]. In this preliminary study, we initially analyzed micro-grafts obtained from human and murine auricular cartilage tissues with in vitro tests in order to characterize the population of cells obtained by the mechanical disaggregation. Secondly, we described the clinical and functional outcomes of cartilage autologous micro-grafting obtained with the Rigenera^®^ protocol in a patient affected by external and internal nasal valve collapse, so-called pinched nose deformity.

## 2. Results

### 2.1. Chondrocyte Murine Micro-Grafts Analysis

**Figure 1:**
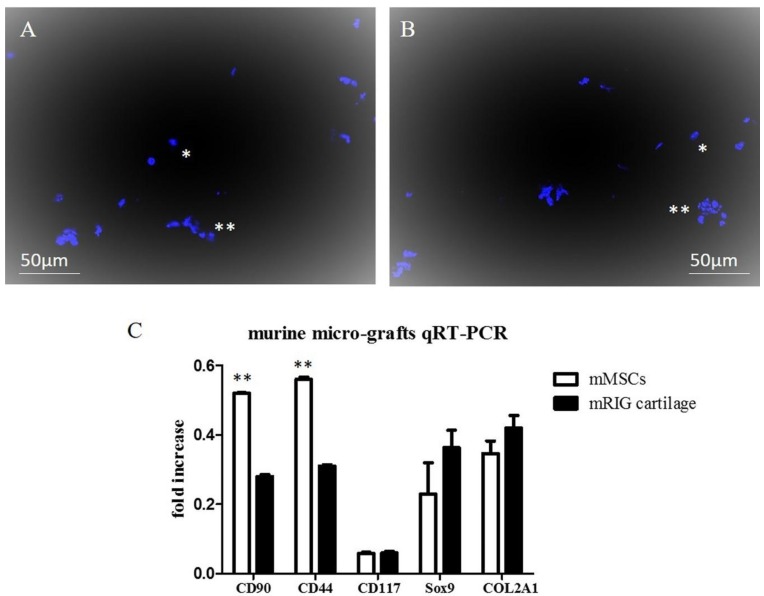
(**A**,**B**): Hoechst 33342 staining on micro-grafts suspension obtained from murine auricular cartilage. (*) represents single nuclei cells, while (**) represents clusters of micro-grafts containing small groups of living cells. Magnification at 20×. (**C**): Gene expression analysis of the indicated specific markers as determined by qRT-PCR. qRT-PCR analyses were performed on murine MSCs cultivated in proliferative medium for 7 days and on micro-grafts obtained from murine auricular cartilage. The graph shows the fold induction of gene expression expressed in arbitrary units. Statistical significance values are indicated as *: *p* < 0.05, **: *p* < 0.01.

Hoechst 33342 staining of suspension obtained with Rigenera^®^ protocol from murine auricular cartilage showed a population of single cells and clusters of micro-grafts containing small groups of intact cells. Morphology and brightness of nuclei together with the ability of the dye to pass the cell membranes indicate that the majority of cells were living cells (Figure 1A,B). The single cell portion of the suspension was found positive by FACS (fluorescence-activated cell sorting) analysis for surface markers CD44 (25%), CD90 (30%), and CD117 (5%), and concomitantly negative for hematopoietic marker CD45 (<2%), thus confirming their mesenchymal origin. RNA from the micro-grafts was quantified (34.5 ng/μL) and subsequently analyzed with quantitative real-rime PCR (qRT-PCR) (Figure 1C). Our results indicate that murine micro-grafts expressed CD44, CD90, and CD117 surface genes, even if their levels were lower with respect to murine MSCs, thus indicating the mesenchymal phenotype of the micro-grafts. In addition, cartilage murine micro-grafts showed the expression of Sox-9 and Collagen II (COL2A1) genes, key genes involved in articular cartilage differentiation. In fact, Sox-9 is identified as a regulator of the chondrocyte lineage [27], while COL2A1 encodes the alpha-1 chain of type II collagen, a fibrillary collagen found in cartilage [28]. The expression of Sox-9 and COL2A1 were expressed at the same level with respect to murine MSCs in proliferative medium, indicating that the micro-grafts obtained from auricular cartilage contained chondrocyte-progenitor cells with a mesenchymal genotype (Figure 1C).

### 2.2. Chondrocyte Human Micro-Grafts Analysis

**Figure 2:**
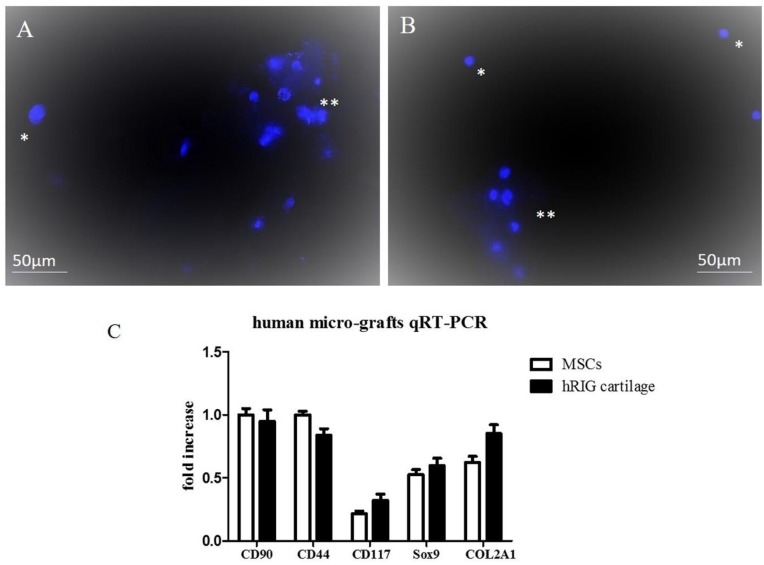
(**A,B**): Hoechst 33342 staining on micro-grafts suspension obtained from human auricular cartilage. (*) represents single nuclei cells, while (**) represents clusters of micro-grafts containing small groups of living cells. Magnification at 20×. (**C**): Gene expression analysis of the indicated specific markers as determined by qRT-PCR. qRT-PCR analyses were performed on human MSCs cultivated in proliferative medium for 7 days and on micro-grafts obtained from auricular cartilage. The graph shows the fold induction of gene expression expressed in arbitrary units. Statistical significance values are indicated as *: *p* < 0.05, **: *p* < 0.01.

Hoechst 33342 staining of suspension obtained with the Rigenera^®^ protocol from human auricular cartilage showed similar results to murine micro-grafts; in fact, single cells and clusters of micro-grafts were observed as in murine Hoechst staining (Figure 2A,B), indicating that the majority of the cells were living cells. FACS analysis demonstrated that human cartilage micro-grafts were positive for surface markers CD44 (35%), CD90 (45%), and CD117 (5%), and concomitantly negative for hematopoietic marker CD45 (<2%). Total RNA from the human micro-grafts was quantified (38.5 ng/μL) and analyzed with qRT-PCR. Results displayed that the cartilage micro-grafts expressed in a similar manner mesenchymal genes (CD90, CD44 and CD117) with respect to MSCs, and also tissue cartilage markers, such as Sox-9 and COL2A1 (Figure 2C).

### 2.3. In Vivo Micro-Grafts

The aim of Rigeneracons^®^ was to disaggregate a small piece of tissue (septum cartilage strips) and opportunely select a cell population with a size of 50 μm. Chondrocytes obtained were suitable to form autologous micro-grafts, which could be used alone or in combination with Platelet Rich Plasma (PRP) as published [29,30], and as reported in a patient analyzed in this work, to obtain a biocomplex ready to be implantable in subjects in need of such intervention. PRP was prepared with the approval of the transfusional service from a small volume of blood. The composite graft obtained (suspension of chondrocytes mixed into PRP in solid form), was applied on the external nasal valve collapse, in the alar cartilage side by fixing with absorbable stitches, improving the soft tissue volume in the site of defects. This construct, subcutaneously injected, resulted in a persistent cartilage tissue with appropriate morphology, adequate central nutritional perfusion without central necrosis or ossification, and further augmented nasal dorsum without obvious contraction and deformation. In addition, microscopic analysis of excisional fragments showed the persistence of healthy cartilage tissue with the formation of new capillaries penetrating into the cartilage as previously reported [29,30]. Here, we demonstrated that the Rigenera^®^ System is a useful method to isolate human chondrocytes when cells were injected with PRP in vivo in patients affected by nasal valve collapse and pinched nose deformity or cartilage defects.

### 2.4. Patients and Clinical Procedure

**Figure 3:**
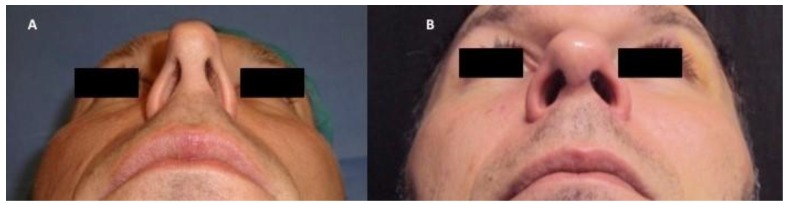
(**A**) Pre-operative situation of patient affected by bilateral external and internal nasal valve collapse with nasal obstruction produced by excessive resection during previous surgery. (**B**) Post-operative situation after 12 months of the same patient treated with condrocyte micro-grafts obtained by Rigeneracons^®^ (CE certified Class I) mixed with platelet-rich plasma (PRP) in solid form. The authors used septum cartilage cut in the strips, processed by Rigenera^®^ Centrifuge. These composite grafts provided functional support to the alar cartilages. Trans-columellar open-tip access was necessary to allow for better visualization of the valve collaspe, alar cartilage, and for the fixation of the cartila.

**Figure 4:**
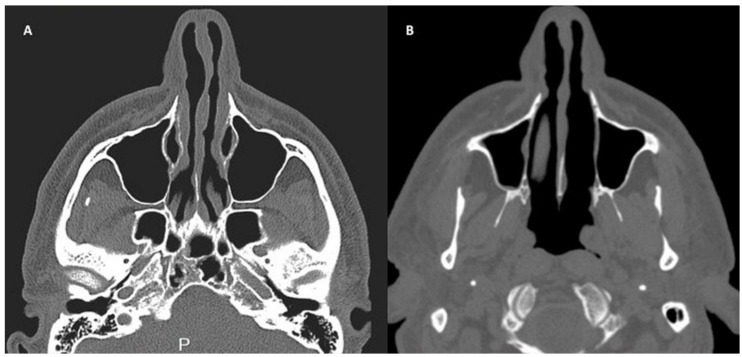
(**A**) CT scans show the pre-operative situation with bilateral soft tissue defect of nasal tip and cartilage collapse. In addition, nasal septum deviation was detected. (**B**) CT scans of the same area after 12 months show the regenerated site in the post-operative image with soft tissue volume improvement and the correction of the nasal septum deviation.

A patient affected by pinched nose deformity (Figure 3A), selected by a group of study treated at the department of Plastic and Reconstructive Surgery of University of Rome “Tor Vergata”, Italy, with chondrocyte micro-grafts gently poured onto PRP in solid form was analyzed. Postoperative follow-up evaluation has shown optimal aesthetic results (Figure 3B) and the improvement of nasal obstruction. These composite grafts provide functional support to the alar cartilages, usually collapsed because of excessive resection during previous surgery. Trans-columellar open-tip access was necessary to allow for better visualization of the valve collapse, alar cartilage, and for the fixation of the cartilaginous structures, to allow for the placement of unexposed absorbable stitches. In the CT (Computed Tomography) scans, the pre-operative situation (Figure 4A) is shown in comparison with the regenerated site in the post-operative image. In particular, CT scans performed after 12 months (Figure 4B) show soft tissue volume improvement and the correction of the nasal septum deviation.

## 3. Discussion

Chondral injuries are a challenging scenario for surgical reconstruction. In particular, nasal valve collapse caused by aging, iatrogenic causes, congenital or secondary injuries after trauma is associated with airway obstruction and significant effects on patient’s life. Despite the good results observed with surgical and prosthetic resurfacing, there is increasing interest in developing safe and effective procedures for biologic resurfacing of cartilage tissues, thanks to the important knowledge of regenerative medicine [16,17,18]. Moreover, articular cartilage is an avascular structure and for this reason exhibits significant problems to repair. Regenerative-tissue engineering approaches combines chondrogenic cells and scaffolds [31]. Regarding chondrocytes, they are isolated from different cartilage tissues, such as articular cartilage, nasal septum, ribs, or ear cartilage [32]. Nevertheless, it is very difficult to grow chondrocytes in vitro, because of their instability in monolayer culture that also causes chondrogenic phenotype loss. Regarding scaffolds for cartilage repair, collagen-based matrices or collagen sponges are the most-used biomaterials [33]. Other scaffolds employed for cartilage repair include agarose, alginate, hyaluronic acid, or cellulose alone or in combination with artificial matrices composed by polylactic acid (PLA) and polyglycolic acid (PGA) or hydrogel [34]. Several techniques have been developed to colonize organic and inorganic polymers, such as cell electrospinning. These jet-based techniques represent a high-resolution method for the manipulation of single cells in order to create biocompatible scaffolds as composite microthreads [20,21]. Frequently, these bio-complexes composed by scaffolds and cells are enriched with platelet-rich plasma (PRP), an autologous concentrated cocktail of growth factors and inflammatory mediators that seems to have a strong positive effect on chondrocyte proliferation in vitro [35]. It has been demonstrated that when adult chondrocytes are cultured in alginate beads in the presence of 10% PRP, the chondrocytes proliferation is higher with respect to chondrocytes grown in classical proliferation medium [35]. However, these different cell-based strategies have not given positive results, probably because their protocols are still far from regenerating a functional and stable cartilage tissue. In addition, many studies with these technologies have passed all in vitro and in vivo mouse models, but they are not clinical-grade methods yet.

The rationale behind the Rigenera^®^ protocol lies in the multipotent properties of mesenchymal stem cells, which may promote cell differentiation and subsequent new cartilage formation [23,24,25]. The Rigenera^®^ protocol opens new surgical strategies especially for tissue repair. Using the Rigenera^®^ protocol, in just one surgical time, the patient is the donor and the acceptor of calibrated micro-grafts. Several authors demonstrated that: (i) Rigenera^®^ improves the healing of bone lesions as suggested by the use of micro-grafts obtained by periosteum in combination with collagen sponge [36]; (ii) Rigenera^®^ is effective in the process of wound healing, improving the superficialization of the wound and reducing wound size [25]; (iii) Rigenera^®^ opens new surgical strategies for rhinoplasty [29,30]. In this manuscript, we demonstrated with in vitro tests that micro-grafts isolated from murine and human auricular cartilage consisted of living single cells and clusters with mesenchymal properties. In addition, gene expression profiling showed similarities between human and murine micro-grafts obtained with the Rigenera^®^ protocol at the transcriptional level, thus confirming their mesenchymal and chondrogenic origins. In order to understand and manage the results, we should consider the ecto-mesenchymal origin of the cranial-facial cartilage samples used in the study. The ecto-mesenchymal origin makes the head cartilage different from the other collectable cartilage tissues in the rest of the body, and this is recognizable in the perichondrium layer maintaining vascularization and a high number of progenitors, mechanically isolated by Rigenera^®^. In fact, in vivo treatment of a patient with pinched nose deformity with chondrocyte micro-grafts isolated from perichondria cartilage during open-tip rhinoplasty, gently poured onto PRP, showed soft tissue volume improvement and the correction of the nasal septum deviation.

In summary, our experience shows that the transfer of autologous micro-grafts using the Rigenera^®^ protocol is safe and has promising results in the treatment of cartilage defects, such as pinched nose deformity. These results suggest the suitability of further prospective, controlled studies with larger cohorts to confirm the therapeutic properties of this intervention. Finally, the Rigenera^®^ protocol could be potentially used in others clinical and regenerative medicine fields, including plastic surgery and orthopedics.

## 4. Materials and Methods

### 4.1. Study Subjects

A 45-year-old male patient affected by pinched nose deformity, selected by a group of study treated at department of Plastic and Reconstructive Surgery of University of Rome “Tor Vergata”, Italy, underwent bilateral nasal alar reconstruction with chondrocyte micro-grafts gently poured onto PRP gel in solid form. The preoperative study included a complete clinical examination of the nasal pyramid and of the nasal cavities through anterior rhinoscopy, a photographic examination in three projections, and computed tomographic scans (CT). Post-operative evaluation included a clinical and a photographic examination, and at a 12-month follow-up, CT scans were conducted. Informed written consent was obtained from patients in order to use their data for research purpose. In particular, nasal obstruction was very marked in the patient analyzed; external nose analysis showed pinched nose deformity, external nasal valve collapse, and a deficit of supra-tip projection (Figure 4A). Anterior rhinoscopy and CT scans (Figure 3A) showed a left deviation of the nasal septum and stenosis of the internal valves with nasal obstruction produced by excessive resection during previous rhinoplasty.

### 4.2. Analysis of Micro-Grafts from Auricular Murine Cartilage

This study was conducted under EU guidelines for the care and use of laboratory animals in accordance with Italian and European legislation (D.lgs. 116/92, European Directives 86/609/EE for the protection of animals used in scientific and experimental studies and 2010-63UE) and was approved by the Ethical Committee recognized from University of Pavia.

Micro-grafts of murine auricular cartilage were obtained by Rigenera Protocol^®^ (CE certified Class I). Auricular cartilage from two ears of C57BL/6 mouse were cut in strips (2 mm × 0.5 mm). The strips were subsequently cut in smaller pieces and dissociated using the Rigeneracons^®^ System (HBW srl, Turin, Italy) in 1.2 mL of physiological solution. After one minute of disaggregation at 80 RPM, the suspension was harvested from the system, filtered through a 40-µm strainer and processed for FACS analysis, Hoechst staining, and RNA extraction. Cytofluorimetric analysis was performed as previously described [37] with some modifications. Briefly, micro-grafts suspension was resuspended in 0.5% BSA in PBS (FACS buffer cells) and incubated with FITC-PE monoclonal antibodies for the following CD antigens: CD44, CD117, and CD45 for 30 min at RT. Analysis was performed on a FACScan (BD Biosciences, San Jose, CA, USA). In addition, micro-grafts were centrifuged at 1200 RPM and stained with Hoechst 33342 (2 μg/mL) for nuclei labeling and samples were observed with a confocal fluorescence microscope (Nikon Eclipse80, ViCo, Nikon, Tokyo, Japan). Hoechst staining was normally used to determine the number of nuclei and to assess gross cell morphology.

Total RNA from micro-grafts was extracted by Trizol^®^ reagent (Invitrogen), according to the manufacturer’s instructions, and retrotranscribed into cDNA with the iScript cDNA Synthesis Kit (BioRad Laboratories) as previously reported [38]. Quantitative reverse-transcription polymerase chain reaction (qRT-PCR) analysis was performed in a 48-well optical reaction plate using a MiniOpticon Real-Time PCR System (BioRad Laboratories). Oligonucleotide primers were designed with gene sequences published in GenBank and are indicated in Table 1. Gene expression was normalized to the phosphoglycerate kinase (PGK) housekeeping gene expression. Each sample was analyzed in triplicate and correlated against a standard curve.

### 4.3. Analysis of Micro-Grafts from Auricular Human Cartilage

Six perichondrial tissue samples surrounding healthy ear cartilage were collected during routine surgeries and cut in strips (3 mm × 0.5 mm). The strips were subsequently cut in smaller pieces and dissociated using Rigeneracons^®^ System (HBW srl, Turin, Italy) in 1.5 mL of physiological solution. After one minute of disaggregation at 80 RPM, the suspension was harvested from the system, filtered through a 40-μm strainer and processed for the same analysis of murine ear cartilage (FACS, Hoechst staining, and molecular gene expression). Oligonucleotide primers were designed with gene sequences published in GenBank and are indicated in Table 1. Gene expression was normalized to the glyceraldehyde-3-phosphate dehydrogenase (GAPDH) housekeeping gene expression. Protocols for gene expression analysis and characterization were the same as those for mouse micro-grafts cartilage.

### 4.4. Chondrocyte Micro-Grafts Preparation for in Vivo Treatment

Autologous micro-grafts of chondrocytes for immediate clinical use were prepared using a medical device called Rigeneracons^®^ (CE certified Class I, HBW srl; Turin, Italy). After the extraction of the perichondrial cartilage during open-tip rhinoplasty, the tissue was cut into strips (2 × 2 mm). The strips were gently collected and disaggregated under sterile conditions (vertical laminar flow hood) by Rigeneracons^®^ (HBW srl; Turin, Italy) in 1.2 mL of physiologic solution. After 60 s of centrifugation at 80 RPM per minute, the cell suspension was collected from the system and gently infiltrated onto PRP gel in solid form. Autologous PRP was performed with specific protocol and was centrifuged, during the same procedure, according to the transfusional service, harvesting a small volume of blood from the peripheral vein of the patient. The product obtained (PRP in solid form mixed with chondrocytes) was applied on the defect in external nasal valve collapse.

## Figures and Tables

**Table 1:** Primers used for qRT-PCR on murine/human cartilage micro-grafts.

Genes	Forward Sequences	Reverse Sequences
mCD90	5′ AAG TCG GAA CTC TTG GCA CC 3′	5′ CCA GGC GAA GGT TTT GGT TC 3′
mCD44	5′ CGA ACC AGG ACA GTG GAG TG 3′	5′ TCT GCC CAC ACC TTC TCC TAC TAT 3′
mCD117	5′ TGA ACG GTA ACA TGG CTG CAT T 3′	5′ ACC ACC GTA AAT GTG TCC CC 3′
mSOX-9	5′ AGA CTC ACA TCT CTC CTA ATG CT 3′	5′ ACG TCG GTT TTG GGA GTG G 3′
mCOL2A1	5′ GGC TCC CAA CAC CGC TAA C 3′	5′ GAT GTT CTG GGA GCC CTC AGT 3′
*mPGK	5′ CAA AAT GTC GCT TTC CAA CAA G 3′	5′ AAC GTT GAA GTC CAC CCT CAT C 3′
hCD90	5′ CAG CGG AAG ACC CCA GT 3′	5′ CGT TAG GCT GGT CAC CTT CT 3′
hCD44	5′ TTA CAG CCT CAG CAG AGC AC 3′	5′ TGA CCT AAG ACG GAG GGA GG 3′
hCD117	5′ GCA CAA TGG CAC GGT TGA AT 3′	5′ GGT GTG GGG ATG GAT TTG CT 3′
hSOX-9	5′ AGG AGA ACC CCA AGA TGC AC 3′	5′ GAG GCG TTT TGC TTC GTC AA 3′
hCOL2A1	5′ AGG ACT GAC CAA GAT GGG AA 3′	5′ AGG GGA GCT GGC TAC TTC TC 3′
*hGAPDH	5′ AGC CTC AAG ATC ATC AGC AAT GCC 3′	5′ TGT GGT CAT GAG TCC TTC CAC GAT 3′
